# Arsenic in Irritative Dyspepsia

**Published:** 1871

**Authors:** 


Arsenic in Irritative Dyspepsia.—Dr. J. C. Thorowgood, in the *Practitioner*, speaks highly of the action of arsenic in many diseases of the stomach. He has found that one-drop doses of Fowler's solution in half an ounce of infus. calumbæ had the effect, in a case he treated, to allay the pain, to stop the vomiting of the food, and to enable the patient to eat and digest small quantities of mutton. He states that the small irritable tongue, with projecting papillæ and yellow or gray fur, indicate arsenic. The more purely local the gastric symptoms, thc better is the chance of arsenic doing good.

When there is much general exhaustion of system, with disordered urine or hepatic congestion, it does not promise much.

[We can confirm this account of the good effect of arsenic in irritative dyspepsia.—Ed. *Edin. Med. Jour.*]

